# Molecular mechanisms and clinical management of cancer bone metastasis

**DOI:** 10.1038/s41413-020-00105-1

**Published:** 2020-07-29

**Authors:** Manni Wang, Fan Xia, Yuquan Wei, Xiawei Wei

**Affiliations:** 1 Laboratory of Aging Research and Cancer Drug Targets, State Key Laboratory of Biotherapy and Cancer Center, National Clinical Research Center for Geriatrics, West China Hospital, Sichuan University, No. 17, Block 3, Southern Renmin Road, Chengdu, 610041 Sichuan P.R. China; 2 Department of Neurosurgery, West China Hospital, Sichuan University, Chengdu, 610041 Sichuan P.R. China

**Keywords:** Bone cancer, Pathogenesis, Bone cancer

## Abstract

As one of the most common metastatic sites of malignancies, bone has a unique microenvironment that allows metastatic tumor cells to grow and flourish. The fenestrated capillaries in the bone, bone matrix, and bone cells, including osteoblasts and osteoclasts, together maintain the homeostasis of the bone microenvironment. In contrast, tumor-derived factors act on bone components, leading to subsequent bone resorption or excessive bone formation. The various pathways involved also provide multiple targets for therapeutic strategies against bone metastases. In this review, we summarize the current understanding of the mechanism of bone metastases. Based on the general process of bone metastases, we specifically highlight the complex crosstalk between tumor cells and the bone microenvironment and the current management of cancer bone metastases.

## Introduction

The distant metastasis of cancer cells has long been known to have characteristic preferences.^1,2^ Bone is one of the most common metastatic sites for malignancies, such as breast, prostate, and lung cancer.^3^ Bone metastases can be categorized into osteolytic metastases with bone resorption, osteoblastic metastases with excessive bone formation, and a mixed phenotype of both.^4,5^ According to the “seed and soil” hypothesis, bone metastasis is dependent on the interactions between tumor cells and the bone microenvironment. The preferential colonization of tumor cells to bone partly relies on the fenestrated capillaries in bone, bone matrix, and cells in the bone marrow (BM) stroma, such as osteoblasts and osteoclasts.^6–10^ These components together maintain the homeostasis of the bone microenvironment. Several deleterious complications, such as ostealgia, fractures, serious hypercalcemia, and nerve compression syndromes, occur in bone metastasis.^11^ As recent advances in bone metastasis research have revealed various pathways involved in this process, both in the primary tumor site and in the resident bone microenvironment, we herein describe the current understanding of the mechanism for bone metastases. Based on the general process of bone metastases, we specifically highlight the complex crosstalk between tumor cells and the bone microenvironment.

## Bone microenvironment

Tumor metastasis is a complex process that involves the reciprocal interactions between tumor cells and the bone microenvironment. The preferential tumor metastasis to bone is therefore probably attributed to the bone microenvironment, which corresponds to the “seed and soil” hypothesis described below.^12^ The bone matrix, BM sinusoid capillaries with a fenestrated structure that provides an abundant blood supply, and the cells in the BM stroma such as osteoblasts and osteoclasts all contribute to the bone microenvironment.^7,10^ Therefore, elucidation of the composition of the bone microenvironment and its interaction with cancer cells can help clarify the underlying mechanisms of metastatic organotropism. The constant remodeling of the bone microenvironment is another potential reason for the preference of circulating tumor cells to colonize bones, and the regulation of cytokines and hormones during this process will be discussed herein.

### The premetastatic niche

The premetastatic niche refers to the supportive environment of potential metastatic sites before the arrival of cancer cells, providing a fertile “soil” to facilitate the invasion, localization, survival, and proliferation of the “seeds,” namely, metastatic tumor cells.^12,13^ In recent decades, studies on the selective colonization of cancer cells to bone have been primarily based on the “seed and soil” theory.^14–16^ First proposed by Steven Paget, this theory was based on the autopsy analysis of 735 breast cancer patients and was published in the *Lance*t in 1889. Intrinsic differences, such as the genomic composition of tumor cells, are primarily attributed to the proliferative phenotype and metastatic potential of the “seeds,” but the microenvironmental condition of the host tissues, or the “soil,” is equally important. The premetastatic niche can be formed even before tumor dissemination due to the tumor-derived factors released from primary tumors.^17^ These factors include several growth factors, such as vascular endothelial growth factor (VEGF). Moreover, the formation of a premetastatic niche relies on a suppressive immune system. Primary tumors recruit myeloid cells, which then allow tumor cells to evade immune surveillance, leading to metastasis.^18,19^ Chemokines facilitate the recruitment of BM-derived cells (BMDCs) and the related immune evasion of primary tumor cells.

### BMDCs

BM is a complex system consisting of multifunctional cells, including hematopoietic and mesenchymal stem cells (MSCs).^20^ The mobilized and recruited BMDCs, as well as other stromal cells, together create a premetastatic niche by releasing various growth factors, inflammatory cytokines and chemokines, and proangiogenic molecules to support tumor cell colonization.^21,22^ Recent studies have identified a subset of nonmalignant BM-derived hematopoietic progenitor cells (HPCs) that express VEGF receptor-1 (VEGFR-1) and promote the arrival of metastatic tumor cells at distant sites.^23^ In response to primary tumor-derived chemokines, VEGFR-1 + BMDCs proliferate in the bloodstream and then preferentially localize to fibronectin-rich areas. At distant premetastatic sites, VEGFR-1 + HPCs upregulate the expression of the very late antigen-4 ligand, which specifically adheres to the newly synthesized fibronectin to establish a fibronectin-rich local microenvironment for cellular cluster formation.^13^ These studies revealed that BM-derived HPCs could help prepare the premetastatic site for tumor metastasis. VEGFR-1 + BMDCs, fibronectin, and associated stromal cells also promote the secretion of other chemokines, such as stromal cell-derived factor-1 (SDF-1), and altogether reshape the bone microenvironment for the colonization, survival, and growth of metastatic tumor cells.^23,24^

### Osteocytes

In addition to osteoblasts and osteoclasts, which we will describe later in this review, osteocytes are a major cell type involved in the regulation of bone modeling and remodeling.^25^ Osteocytes are abundant in the calcified bone matrix and have a unique structure that allows them to interconnect with each other, osteoclasts, and BM cells through dendritic processes.^26^ This highly interconnected network allows the exchange of nutrients and metabolites of bone cells and the transport of assorted factors from osteocytes. Factors produced by osteocytes include sclerostin (SOST)^27^ as well as receptor activator of nuclear factor-kappa B ligand (RANKL),^28,29^ dentin matrix acidic phosphoprotein 1,^30^ and β-catenin,^31^ which modulate bone formation and resorption, especially in response to mechanical stimuli. Osteocytes also act as an endocrine organ that releases soluble growth factors to regulate the physiological functions of distant organs such as the kidney^32^ for the maintenance of phosphate and vitamin D equilibrium.^33^

### BM MSCs

MSCs are multifunctional non-HPCs. BM-derived MSCs (BMSCs) are a crucial MSC subgroup for osteogenesis and chondrogenesis.^34^ Due to their potent capacity for differentiation, BMSCs develop into cells such as BM stromal cells, skeletal myocytes, and osteoblasts.^35–37^ Recently, BMSCs have been shown to display immunoregulatory properties.^38,39^ The BM microenvironment is potentially conducive to the development of T cells in the absence of the thymus.^40,41^ However, prior studies have indicated that BMSCs can inhibit the proliferation of mature T cells and natural killer cells.^42,43^

### Regulatory factors in the premetastatic niche

Compelling preclinical data have indicated that even before tumor cell migration, primary tumors can release soluble molecules into the circulation and prepare the soil for disseminating tumor cells. Exosomes are small vesicles of ~100 nm in diameter that are released by cells.^44^ Exosomes secreted by cancer cells have recently been found to express integrins, a group of membrane receptors that allow the targeted movement of exosomes toward distant organs.^45^ For example, exosomes expressing integrin αvβ5 preferentially move to the liver, while those expressing α6β4 target the lungs.^45^ The secreted exosomes are then internalized by the host organ cells, as α6β4-expressing exosomes colocalize with S100A4-positive fibroblasts in the lung and integrin αvβ5-expressing exosomes colocalize with S100P-positive Kupffer cells in the liver.^45^ S100A4 and S100P belong to the S100 family, a group of acidic Ca^2+^-binding proteins that interact with various intracellular effector proteins and mediate downstream protein phosphorylation and cytoskeleton dynamics.^46,47^ The expression of S100A8/A9 is substantially elevated in human breast cancer cells, resulting in a migratory phenotype in cancer cells.^48^

The best-characterized function of lysyl oxidase (LOX) is extracellular matrix (ECM) remodeling, which by strengthening the crosslinking of collagen and elastin, thereby improving the structural integrity of the ECM.^49^ LOX expression can be induced under hypoxia, which is frequently observed in almost all solid tumors, and LOX can thus be used as a biomarker for premalignant changes during tumorigenesis.^50^ Moreover, LOX can prepare the premetastatic niche by activating bone resorption.^51,52^ Bone homeostasis is partly regulated by LOX activity, either directly through an RANKL-independent pathway BM stromal cells such as osteoblasts and osteoclasts^52^ or indirectly through RANKL-dependent mechanisms.^53^ In these ways, LOX disrupts bone homeostasis and facilitates premetastatic lesion formation. The elevated collagen fiber reticulation,^54^ which facilitates the anchorage and colonization of neoplasms in metastatic sites,^51,54^ is probably the cause of the protumoral effects of LOX. A recent investigation indicated that LOX silencing in primary tumors or antagonism of tumor-secreted LOX can prevent the formation of focal premetastatic lesions and the subsequent metastatic burden in bones.^55^ Prior studies have also found the existence of exosomes secreted by bone cells in the bone microenvironment.^56–58^ Exosomes can either be derived from osteoclasts to regulate osteoclastogenesis^59^ or released from osteoblasts to stimulate RANK signaling in osteoclast precursors, ultimately inducing osteoclast formation.^60^ One such example is exosomal-derived osteoclastic miR-214-3p, which is significantly correlated with bone formation in elderly female individuals with bone fractures.^61^

The hypoxic bone microenvironment could promote cancer cell metastasis and growth. A key mediator of hypoxic signaling is hypoxia-inducible factor-1 (HIF-1), which initiates the transcription of hypoxia-response-related genes.^62^ Many of these genes are prometastatic and are essential for angiogenesis, tumor cell apoptosis, and growth factor/cytokine activities.^63^ Tumor cells that can survive in the hypoxic bone microenvironment then colonize and thrive in bones, leading to a vicious cycle of bone metastases. HIF-1α interacts with several growth factors and cytokines, such as VEGF, insulin-like growth factors (IGFs), fibroblast growth factors (FGFs), and epidermal growth factor; the expression of these molecules on cancer cells can further enhance cancer cell proliferation and metastasis.

Extracellular pH in bones is associated with osteoblast and osteoclast function and with bone acidification leading to enhanced osteoclast resorption.^64^ Metastatic tumor cells in localized bone regions produce lactic acid, resulting in acidosis of the bone microenvironment.^64^ Tumor acidosis, in turn, increases the secretion of proteins that degrade the ECM and thus facilitate metastases, such as cathepsin B and matrix metallopeptidases (MMPs).^65^

## Osteolytic and osteoblastic bone metastasis

**Fig. 1:**
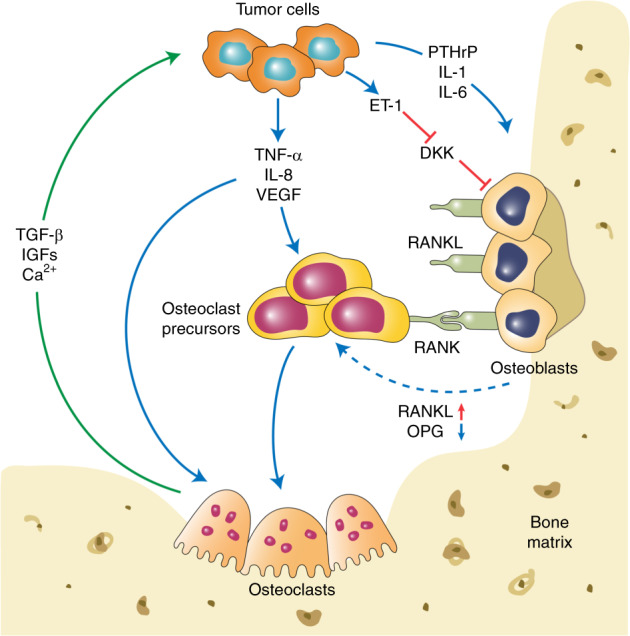
The development of osteoclastic and osteoblastic bone metastases. Tumor cells interact with both osteoclasts and osteoblasts in the bone microenvironment, which leads to a local increase in tumor-derived factors to promote osteoclastogenesis and osteoblastogenesis. Mature osteoclasts, in turn, release survival factors, such as insulin-like growth factor 1 (IGF-1) and transforming growth factor beta (TGF-β), which promote the survival and proliferation of tumor cells

The two major categories of bone metastases are osteolytic and osteoblastic, based on which type of cells exhibit the predominant activities,^66^ and the impaired balance between bone formation and resorption is frequently observed in both types of metastasis. Despite the excess occurrence of bone resorption and formation, growing evidence has suggested the coexistence of osteolytic and osteoblastic metastases, leading to mixed-type bone metastases.^67^ Based on the distinct cytokine profile detected, lung cancer-derived bone metastases are preponderantly osteolytic,^68^ whereas prostate cancer shows preferential osteoblastic bone metastases.^69^ The development of osteoclastic and osteoblastic bone metastases is shown in Fig. 1.

### Osteolytic bone metastasis

One prerequisite for the occurrence of osteolytic bone metastases is osteoclast activation. Osteoclasts are multinucleated cells that differentiate from their mononuclear macrophage/monocyte-lineage hematopoietic precursors and are involved in regulating intracellular calcium and inorganic phosphate levels. In the BM, multiple osteoclastogenic factors induce the differentiation of mononuclear macrophage/monocyte-lineage hematopoietic precursors into osteoclast precursors, which enter the bloodstream and localize to the remodeling sites of the bone.^70^ The differentiation of osteoclast precursors is initiated after exposure to the two main regulatory factors, macrophage colony-stimulating factor and RANKL. RANKL, produced by osteoblasts, binds to its receptor RANK on the osteoclast precursor surface, which stimulates downstream signaling molecules, including mitogen-activated protein kinases (MAPKs) and phosphatidylinositol 3-kinase (PI3K)/Akt, and promotes the maturation of osteoclast precursors into functional osteoclasts.^71^ In addition to RANKL, osteoprotegerin (OPG), a decoy receptor that is produced by osteoblasts, eliminates RANKL and thus inhibits the RANK–RANKL signaling pathway. Therefore, the activation of osteoclasts is attributed to the delicate balance between RANKL and OPG.^72,73^

#### RANK/RANKL

RANK is a surface receptor of the tumor necrosis factor (TNF) family.^74^ This receptor is crucial for the formation, activation, and function of osteoclasts and also regulates calcium metabolism.^75^ Although RANK is considered to be primarily expressed on osteoclasts and their progenitors, recent studies have also detected its expression on tumor cells, indicating the potential participation of RANK in tumor metastasis.^76,77^ RANKL is a polypeptide that belongs to type II transmembrane proteins.^78^ RANKL, the ligand of RANK, can be expressed on the surface of osteoblasts and bone stromal cells and exists within the bone microenvironment in a soluble form. Recent investigations have also detected high expression of RANKL in osteocytes, suggesting an important role in bone remodeling.^28,29^ Moreover, RANKL has been found on both T and B lymphocytes. However, this molecule is not involved in bone remodeling under normal conditions.^28^ Several osteotropic factors, including parathyroid hormone (PTH), vitamin D3, TNF-α, Wnt Family Member 5A, and IL-6, can stimulate RANKL expression, thus promoting osteoclastogenesis.^79,80^ In osteoclast precursor cells, RANKL enhances the production of mature osteoclasts through stimulation of M-CSF at low levels.^81,82^

RANK/RANKL signaling involves many transcription factors. The recruitment of TNF receptor-associated factors (TRAFs) is crucial for RANK/RANKL-mediated osteoclastogenesis, which activates various transcription factors, such as nuclear factor kappa beta and AP-1, and prevents the apoptosis of mature osteoclasts.^83,84^ RANKL also induces the phosphorylation of another transcription factor, microphthalmia transcription factor (MITF), and activates downstream MAPK.^85^ The stimulated transcription factor complex has a functional role in the expression of osteoclast-specific genes. The cytoplasmic domain of RANK recruits the adapter proteins Gab2 and PLCγ2 and thus activates calcium signaling in osteoclastogenesis.^86^ PLCγ2 then acts synergistically with costimulatory signals to activate NFATc1, a transcription factor regulated by calcium signaling.^87,88^ Furthermore, NFATc1 participates in the transcription of vacuolar ATPase and dendritic cell-specific transmembrane protein, which are closely related to the multinucleation of osteoclasts.^89–91^

In a breast cancer model, RANKL exerts its promigratory effect on cancer cells and thus promotes their metastasis to bone.^92^ Although breast cancer cells do not produce RANKL, they produce parathyroid hormone-related protein (PTHrP), which stimulates RANKL production in bone cells.^93–95^ PTHrP regulates the activation of osteoclasts as a specific mediator of osteolysis in breast cancer metastases.^93,96^ The expression of PTH-rP was significantly higher in breast cancer cells that metastasize to the bone than in those in nonbone soft tissues.^97–99^ Moreover, monoclonal antibodies (mAb) that target PTH-rP potently inhibited the progression of bone metastases.^100^ Hence, PTH-rP can promote bone metastases by activating the bone resorption activity of osteoclasts.

#### OPG

Conceptually, the balance between RANKL and OPG activities primarily determines the level of osteoclastogenesis, with a relatively higher OPG level leading to decreased bone resorption.^101,102^ OPG is a member of the small integrin-binding ligand N-linked glycoprotein family and is a soluble receptor specific for RANKL.^103^ In this way, OPG competes with RANK for RANKL and thus hinders RANKL–RANK communication on the osteoclast cell membrane and disrupts osteoclastogenesis and subsequent bone resorption.^104^ However, some ECM components within bone microenvironments, such as glycosaminoglycans, may inhibit the RANKL–OPG interaction.^105,106^

In prostate cancers, the overexpression of OPG could inhibit the development of bone metastatic tumor cells, with no impact on the proliferation of tumor cells.^107^ Researchers have hypothesized that the essential role of osteolysis in tumor-bone metastases is to release growth factors from bones and to maintain the space needed for tumor growth in bone. In this indirect manner, OPG prevents bone lysis and thus reduces metastatic bone lesions. Previous reports have emphasized that the OPG produced by BM stromal cells was associated with the survival capacity of malignant prostate cells.^108^ As described earlier, OPG can also be produced by cancer cells and protects cancer cells from TNF-related apoptosis-inducing ligand-induced apoptosis in a prostate cancer model.^109^ However, a recent study found that the serum level of OPG was increased in patients with prostate cancer bone metastasis compared with nonmetastatic patients.^110^ These contradictory results indicate the need for further studies on the role of serum OPG level as a bone metastatic marker of prostate cancers.

#### Calcium-sensing receptor (CaSR)

Extracellular calcium released from the bone matrix plays an active role in the vicious cycle of cancer bone metastasis. Under physiological conditions, the calcium balance is delicately maintained within a physiologic range in the bone microenvironment. The extracellular calcium of cancer cells is recognized through the CaSR or the P2X receptor, which manipulates calcium influx and efflux through ion channels or transporters. Therefore, once cancer cells reach bones, exposure to high calcium concentrations in the microenvironment, in turn, activates the CaSR. Although some evidence indicates that CaSR plays a tumor-suppressive role in gastric and colon cancers,^111,112^ this receptor has also been shown to promote bone metastasis of some other cancer types, such as renal cell carcinoma (RCC), as CaSR is widely detected in both normal and malignant renal tissues.^113^ In a clinical study, higher expression of CaSR was observed in RCC patients with bone metastases than in primary cancer patients 5 years after surgery.^114^ Due to the osteolytic activities in RCC bone metastases, the high serum calcium concentrations may potentially increase the activities of CaSR-expressing tumor cells.^115^ A recent study found that CaSR overexpression increased the adhesion, migration, and proliferation of RCC cells in a calcium-dependent manner, indicating that cellular calcium might enhance the metastatic behavior of RCC via CaSR.^116^ Similar findings were reported in breast cancer cells in which the overexpression of CaSR was correlated with an increase in osteolytic potential.^117^

#### TNF-α

TNF-α, a proinflammatory cytokine, is frequently observed in the tumor microenvironment and is mainly produced by tumor-associated macrophages and tumor cells.^118–120^ TNF-α was reported to accelerate tumor cell apoptosis at a high dose.^121^ However, when released into the tumor microenvironment, TNF-α also promotes cancer metastases at a low dose.^122^ The proinflammatory cytokine TNF-α is one of the strongest inducers of bone resorption.^123^ This molecule can stimulate the expression of RANKL and M-CSF in stromal cells, osteoblasts, and activated T cells^124–127^ and directly promote the formation of TRAP^+^ multinucleated osteoclasts in the presence of M-CSF and the absence of RANKL by activating NF-κB and AP-1 signaling.^128–131^ Moreover, a recent investigation reported the correlation between RANKL and TNF-α in osteoclastogenesis.^132^ TNF-α induces osteoclast differentiation from TRAF6^−/−^ osteoclast precursors through the RANKL-mediated degradation of TRAF3, suggesting that RANKL could enhance TNF-α-induced osteoclastogenesis in a TRAF6-independent manner.^132^ In RA patients, TNF-α upregulates the IL-34 level via the NF-κB and JNK signaling pathways,^133^ and TNF-α inhibitors, such as infliximab, adalimumab, certolizumab, and golimumab, have all shown clinical success.^134^

#### Interleukins (ILs)

IL-1, either directly or indirectly, acts on the differentiation of osteoclasts depending on the levels of other growth factors in the bone microenvironment.^135,136^ For example, IL-1α has been reported to directly induce osteoclast differentiation through MITF in BM macrophages in an RANKL-independent mechanism.^137^ However, the activation of osteoclastic markers, such as TRAP, cathepsin K, and MMP-9, by IL-1α can also be associated with RANKL levels.^138^ IL-1β, a proinflammatory cytokine, can potently stimulate osteoclast differentiation and subsequent bone resorption.^139^ Likewise, IL-1β either indirectly stimulates TNF-α-induced osteoclastogenesis by inducing RANKL expression or directly promotes p38 MAPK-regulated osteoclastogenesis in the presence of sufficient RANKL.^140^ Treatment strategies targeting IL-1, such as IL-1 receptor inhibitors (e.g., anakinra) and IL-1 antagonists (e.g., rilonacept and canakinumab), are now in clinical use for RA patients.^141^

The IL-6 cytokine family members share a common subunit, gp130, in their signaling receptor complex.^142^ IL-6 promotes osteoclastogenesis via interaction with the IL-6 receptor (IL-6R), which induces RANKL expression in osteoblasts and stromal cells.^143^ Interestingly, OPG treatment failed to prevent osteoclastogenesis induced by IL-6 in the presence of M-CSF, which could be inhibited by a gp130 antibody.^144^ Likewise, in an animal model, anti-human IL-6R antibodies could also prevent bone metastases.^145^ Thus, IL-6 may promote osteoclastogenesis in an RANKL-independent manner.^146^ One study, however, has reported the suppressive function of IL-6 in osteoclast differentiation; IL-6 inhibited RANK-mediated NF-κB and JNK activation.^143^ This result was supported by a recent study showing that the IL-6 and IL-6R interaction could differentially manipulate RANKL-induced osteoclastogenesis via the NF-κB, ERK, and JNK signaling pathways.^147^ In the presence of low-level RANKL, IL-6/IL-6R enhanced osteoclastogenesis, which was significantly suppressed by high-level RANKL. Thus, IL-6 can act either as an osteoclastogenic factor or bone protector, depending on the level of RANKL in the bone microenvironment. Oncostatin M (OSM), a member of the IL-6 family, exhibits multiple effects in physiological processes, including hematopoiesis, neurogenesis, and bone homeostasis.^148^ OSM was shown to promote epithelial to mesenchymal transition (EMT)^149^ and tumor cell invasion,^150,151^ as well as upregulate proteases to degrade the local ECM in breast cancer.^151,152^ In an in vitro experiment, breast cancer cell lines cocultured with OSM showed increases in osteoclastogenesis, which could be reversed by treatment with amphiregulin, an antibody targeting a previously uncharacterized OSM-regulated bone metastatic factor.^153^

By increasing the release of RANKL and TNF-α in T cells, IL-7 has previously been identified as an indirect stimulator of osteoclast formation.^154–157^ TNF-α, in turn, stimulates IL-7 production, which promotes the expansion of IL-17-producing T helper 17 (Th17) cells.^126^ The Th17 cytokine IL-17 is an RANKL inducer known to disturb the RANKL/OPG balance and ultimately lead to bone resorption.^158^ A recent investigation, however, indicated that IL-7 could directly induce osteoclastogenesis via STAT5 signaling, which was independent of RANKL.^159^ Other pathways were then identified by the neutralization of IL-17A, which blocked C-X-C chemokine receptor type 4 (CXCR4)/SDF-1 signaling in metastatic microenvironments and substantially decreased bone metastases.^160^ However, the inhibitory effect of IL-17 on osteoclastogenesis, which mainly involves the induction of granulocyte-macrophage colony-stimulating factor (GM-CSF) in osteoblasts, has been observed.^161^ GM-CSF is secreted by cells, including activated T cells, fibroblasts, and macrophages, and then stimulates osteoclastogenesis via Ras/ERK signaling.^162,163^ GM-CSF was shown to increase the number of osteoclast precursors within the bone microenvironment,^164^ which is contradictory to another study suggesting that the expression of TNF-α-induced GM-CSF suppresses hematopoietic precursors of osteoclasts.^165^ Therefore, the role of GM-CSF in osteoclastogenesis remains unclear.

IL-11 has long been identified as a functionally pleiotropic member of the IL-6 cytokine family due to its capacity to stimulate IL-6-dependent cell proliferation.^166^ This molecule is initially produced in a BM-derived stromal cell line and can be released by mature osteoblasts to enhance osteoclastogenesis.^167^ An early study demonstrated that various osteotropic factors, including IL-1, TNFα, PGE2, PTH, and 1 alpha, 25-dihydroxy vitamin D3 [1 alpha, 25(OH)_2_D3], could promote the production of IL-11 by osteoblasts, whereas IL-6, IL-4, and TGF-β could not.^168,169^ In cocultures of both osteoblasts and BM cells, IL-11 induced the formation of osteoclast-like multinucleated cells in a dose-dependent manner, but this process was strongly inhibited by anti-IL-11 antibodies.^170^ A recent clinical study found that the serum levels and mRNA expression of IL-11 in breast cancer patients were significantly elevated in the metastatic group compared with the nonmetastatic group, suggesting that IL-11 has predictive value in breast cancer bone metastasis.^171^

IL-8 is another potential stimulator of osteoclastogenesis and bone destruction in bone metastases.^172^ IL-8 may bind to the IL-8 receptor (IL-8R) on the osteoclast surface independent of RANK–RANKL signaling,^173^ and IL-8 antibodies or IL-8R inhibitors significantly suppressed osteoclast differentiation in vitro.^174^ A recent study suggested that the serum level of IL-8 was elevated in patients with anti-citrullinated protein antibody (ACPA)-positive RA and that ACPA-induced osteoclastogenesis can be inhibited by IL-8 neutralizing antibodies.^175^

### Osteoblastic bone metastasis

Despite recent research efforts on osteoclastic bone metastases, little is known about this condition, which mainly occurs in advanced prostate cancers and less frequently occurs in breast cancers. Osteoblasts are stimulated by metastatic tumor cell-derived factors, including FGFs, urokinase-type plasminogen activator (uPA), endothelin-1 (ET-1), prostate-specific antigen (PSA), IGFs, bone morphogenic proteins (BMPs), and VEGF.^176–180^ ET-1 plays a vital role in the osteoblastic response to cancer bone metastasis.^181^ The binding of ET-1 to the endothelin A receptor (ETAR) downregulates the autocrine production of a Wnt antagonist, Dickkopf-1 (Dkk-1).^182,183^ The subsequent Wnt pathway activation is crucial for the differentiation and function of osteoblasts. Moreover, a previous study reported that the inverse correlation between the level of Dkk-1 and osteoblastogenesis is independent of osteoclastogenesis.^184^

#### ET-1

ET-1, acknowledged as a vasoactive peptide, is actively involved in the formation of new bone in the context of osteoblastic bone metastasis.^185,186^ In a mouse tumor model, the osteoblastic metastasis of breast cancers was closely associated with the secretion of ET-1, the activity of which relied on its binding to ETAR.^181^ The study also demonstrated that ETAR blockade strongly suppressed osteoblastic bone metastasis and reduced the tumor burden in bone, suggesting the potential value of ETAR inhibitors for bone metastatic cancer patients.^181^ However, no precise molecular mechanisms for ET-1-regulated bone metastases have been established. Previous research has indicated that ET-1 can suppress osteoblast apoptosis by stimulating the calcineurin/NFAT pathway.^187^ This process may also involve E-cadherin augmentation, which correlates with tumor cell adhesion, as well as upregulated Runx2 activity and SPARC expression, which is related to osteomimicry.^188,189^ The inductive effect of ET-1 on IL-18 expression has been identified in osteoblasts at the gene promoter/transcriptional level through a p38 MAPK-dependent pathway.^190^ IL-18, as discussed above, acts as a regulator of osteoblast proliferation. A recent study also evaluated the impact of ETS proto-oncogene 1 (ETS-1) and HIFs on ET-1 signaling and found that the balance between ETS-1 and HIF might affect downstream signals represented by ET-1.^191^

#### DKK-1

As discussed earlier in the review, bone metastases usually have a mix of osteolytic and osteoblastic metastases. Accumulating evidence has suggested that osteolysis is the first step, even in the osteoblastic metastasis setting. In prostate cancer bone metastasis, DKK-1 acts as a molecular switch that converts osteolytic metastasis to osteoblastic metastasis.^192^ DKK-1 also enhances the bone metastasis of breast cancers through the regulation of canonical WNT pathways in osteoblasts. However, targeting canonical WNT alone may fail to prevent cancer metastases, whereas combinational inhibition of JNK and TGF-β signaling could effectively treat cancer metastases to the lung and bone.^193^ Cancer cells themselves can also secrete DKK-1 and regulate DKK-1 levels in the local microenvironment independently of ET-1. For example, PC3, a prostate cancer cell line, could produce DKK-1 and was converted from an osteolytic to an osteoblastic phenotype when transfected with Dkk-1 siRNA.^184^ In a clinical setting, the serum levels of DKK-1 were robustly elevated in breast cancer patients with bone metastases compared with healthy controls or nonmetastatic patients.^194^ Another inhibitor of WNT signaling is SOST, the functional loss of which may lead to multiple bone disorders due to dysregulation of bone remodeling.^195,196^ Sclerostin, known as the protein product of the SOST gene, can inhibit canonical WNT signaling^197,198^ and thus promote bone formation.^199^

#### Other factors regulating osteoblastic bone metastasis

The role of PTHrP in osteoblastic metastases, particularly in prostate cancers, has long been disputed.^200^ The expression of PTHrP correlates with increased malignancy and incidence of skeletal metastasis in multiple cancers, including prostate cancers.^201,202^ Tumor-derived PTHrP not only plays an important role in the bone remodeling process but also directly facilitates the proliferation, adhesion, and survival of tumor cells.^203–205^ PTHrP was shown to potently stimulate osteoclastogenesis by increasing the production of RANKL by osteoblasts.^206^ However, PTHrP also facilitates osteoblastic alterations.^207^ PSA, a serine proteinase, can cleave PTHrP into fragments at residue 23,^208^ impairing PTH/PTHrP-mediated activation of its receptor.^209^ Given the structural similarity between PTHrP-1-16 and ET-1 in the N-terminus, the inactive fragment PTHrP-1–16 can bind to and thus activate ETAR.^210^ In addition to its cleavage of PTHrP, PSA may stimulate osteoblasts by preventing IGF from its binding protein and transforming latent TGF-β into its active form.^211,212^ In vivo studies are needed to demonstrate PSA-mediated bone metastasis, as most of the evidence thus far is based on in vitro studies.^213^ Prostate cancer cells also secrete the paracrine factor BMP4, an activator of osteoblast differentiation.^214^ A recent study suggested that BMP4 might potentially mediate the endothelial-to-osteoblast (EC-to-OSB) conversion of endothelial cells into osteoblasts (28586644), which was consistent with previous reports that treatment with a BMP receptor inhibitor in mice with prostate cancer significantly prevented tumor-induced bone formation (21670081). Furthermore, prostate cancer cells produce FGF-9, which promoted the osteoblastic phenotype of MDA PCa-118 xenografts.^215^

## The multistep process of bone metastases

**Fig. 2:**
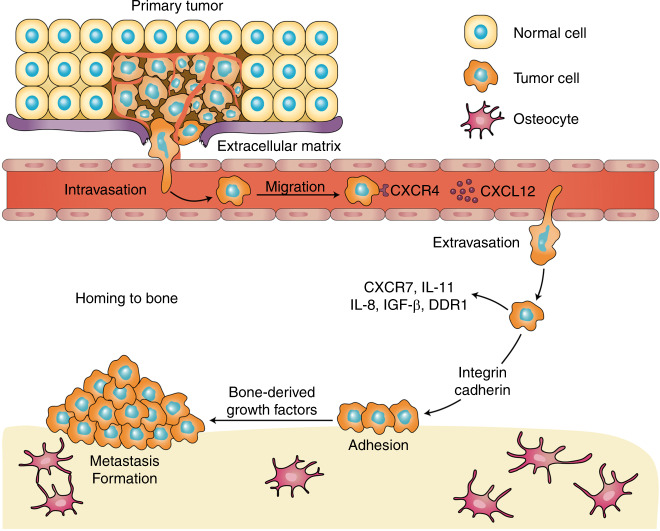
The general multistep process of bone metastasis and its correlated regulatory factors

Bone metastasis does not occur randomly. This process is a well-organized procedure that involves a vicious cycle between the tumor and bone, where one promotes the other, disrupting the bone matrix and leading to bone metastasis.^216,217^ The general bone metastatic process can be divided into cancer cell escape and dissemination, adhesion and invasion to the bone, and metastasis in bone. The general multistep process of bone metastasis and its regulating factors are presented in Fig. 2 and Table 1.

**Table 1:** Regulating factors associated with the bone metastasis process

Regulating factors	Action mechanism
Tumor cell dissemination	
MMP-2	Aacts synergistically with E-cadherin and facilitates tumor angiogenesis
MMP-7	Promotes osteolytic bone metastases by increasing the soluble form of RANKL
MMP-9	Induces tumor angiogenesis
MMP-13	Mediates tumor-induced osteolysis and indicates the invasion and metastasis capacity of tumors
Tumor cell adhesion and invasion	
CXCL12/CXCR4	Facilitates the mobilization of hematopoietic stem cells, tumor proliferation, and angiogenesis
Galectin-3/TF	Promotes adhesion of cancer cells to bone marrow endothelial monolayers
CD44/hyaluronan	Promotes adhesion of cancer cells to bone marrow endothelial monolayers
CCL5/CCR5	Induces tumor cancer cell migration
XCL1/XCR1	Promotes the proliferation and migration of tumor cells
ADAM	Facilitates the degradation of the bone matrix
uPA82	Facilitates the degradation of the bone matrix
COX-2	Facilitates tumor cell adhesion and proliferation in bones
Metastasis formation in bone	
TGFβ	Dual role: suppresses tumor growth at early stage and promotes invasion and metastasis to bones at later stages
IGF-1	Promotes bone colonization of metastasizing tumor cells and facilitates their expansion inside bones
miR-141-3p	Promotes EMT leading to cancer cell invasion and migration
miR-145	Promotes EMT leading to cancer cell invasion and migration
miR-335	Suppresses the development of bone metastases
miR-126	Suppresses the development of bone metastases
miR-206	Suppresses the development of bone metastases
miR-135	Reduces metastasis formations in bone via the downregulation of Runx2
miR-203	Reduces metastasis formations in bone via the downregulation of Runx2
miR-10b	Promotes the bone marrow invasion of tumor cells
miR-21	Promotes the bone marrow invasion of tumor cells
ATX	Regulates the early stage of tumor cell colonization in bones
Osteoclastic metastases	
RANK/RANKL	Activates transcription factor and prevents the apoptosis of mature osteoclasts
PTHrP	Activates the bone resorption activity of osteoclasts
OPG	Competes with RANK for RANKL and thus blocks the RANKL/RANK interaction and osteoclastogenesis
CaSR	Mediates the adhesion, migration and proliferation potential of tumor cells in a calcium-dependent manner
TNF-α	Upregulates the expression of RANKL and induces osteoclast differentiation from TRAF6^−/−^ osteoclast precursors
IL-1	Directly or indirectly promotes osteoclastogenesis by inducing RANKL expression
IL-6	Promotes osteoclastogenesis by inducing RANKL expression in osteoblasts and stromal cells
IL-7	Induces RANKL expression and disturbs the RANKL/OPG balance
GM-CSF	Dual role: stimulates osteoclastogenesis, or suppresses hematopoietic osteoclast precursors
IL-11	Induces the formation of osteoclast-like multinucleated cells
IL-8	Stimulates osteoclastogenesis and bone destruction in metastatic bone diseases
Osteoblastic metastasis	
ET-1	Suppresses osteoblast apoptosis and promotes tumor cell adhesion via E-cadherin augmentation
DKK-1	Converts osteolytic metastasis to osteoblastic metastasis
PTHrP	Participates in the bone remodeling process and facilitates tumor cell proliferation, adhesion and survival

*MMPs* matrix metallopeptidases, *CXCR4* C-X-C chemokine receptor type 4, *TF* Thomsen–Friedenreich, *COX-2* cyclooxygenase-2, *TGF-β* transforming growth factor beta, *IGF* insulin-like growth factor, *miRNA* microRNA, *ATX* autotaxin, *RANL* receptor activator of nuclear factor kappa-B ligand, *PTHrP* parathyroid hormone-related protein, *OPG* osteoprotegerin, *CaSR* calcium-sensing receptor, *TNF* tumor necrosis factor, *GM-CSF* granulocyte-macrophage colony-stimulating factor, *ET-1* endothelin-1, *Dkk-1* Dickkopf-1

### Cancer cell escape and dissemination

The ability of tumor cells to escape their local microenvironment and degrade ECM proteins is an integral part of the malignancy of cancers. To intravasate into the bloodstream and colonize the metastatic site, tumor cells must pass through the basement membrane and the ECM. MMPs are a superfamily of multiple zinc-dependent proteinases that degrade ECM proteins.^218^ High MMP levels have been observed in various malignancies, including prostate, bladder, lung, and breast cancers, as well as head and neck squamous cell carcinomas,^219–222^ and are correlated with poor clinical outcome.^223,224^

The MMP family is closely correlated with angiogenesis. Both in vitro and in vivo investigations have reported the antiangiogenic effect of MMP inhibitors.^225–227^ The angiogenic response was shown to be significantly reduced in MMP-deficient mice.^228,229^ Of all the MMP members, MMP-2 is the best-studied protein due to its function in angiogenesis. The addition of exogenous pro-MMP-2 to endothelial cell culture could lead to morphologic changes that indicate angiogenesis.^230^ Furthermore, MMP-2 acts synergistically with adhesion molecules (e.g., E-cadherin).^231^ High expression of both MMP-2 and MMP-9 (an MMP family member closely related to MMP-2) was linked to a poor prognosis in breast cancer.^224^ In support of this hypothesis, MMP-2 positivity indicated an increase in the risk of death in the first 10-year follow-up.^232^ Furthermore, MMP-2 was substantially elevated in patients with HER2/neu gene-amplified tumors, known as an aggressive tumor phenotype. A previous investigation evaluating the association between MMP-2 and clinicopathological parameters found that MMP-2 was an indicator of more invasive phenotypes and was related to lymph node metastasis.^233^ MMP-2 also induces angiogenesis through the regulation of VEGF and the cleavage of ECM molecules (e.g., type IV collagen)^234,235^ and therefore facilitates angiogenesis in the tumor microenvironment.^236^ However, previous studies have found that MMP-2 promotes the release of bioactive fragments of ECM, such as endostatin,^237^ restin,^238^ and arrestin,^239^ which inhibits angiogenesis. This inhibitory effect is related to the dormancy of breast cancer, where MMP-2 induces disseminated breast tumor cells to enter dormancy by promoting the expression of the dormancy promoter TGF-β2 in the BM.^240^ A recent report found that thrombospondin-2 could promote the migration of prostate cancer cells by enhancing MMP-2 expression.^241^

Osteolytic bone metastasis was significantly reduced in an MMP-7-deficient prostate cancer model, which had low levels of osteolysis due to defects in RANKL processing and osteoclast activation.^242^ MMP-7, producing a soluble form of RANKL from membrane-bound RANKL, promotes osteolytic bone metastases in prostate cancer.^242^ In prostate cancers, tumor growth in the bone microenvironment can be stimulated by osteoclast-derived MMP-9, which enhances angiogenesis without altering the osteolytic or osteogenic properties of tumors.^243^ However, BMP-6, a member of the TGF-β superfamily, suppresses the paracrine secretion of MMP-9 in breast cancer cells via MAPK/p38/AP-1 signaling.^244^ MMP-13 overexpression was first detected in breast carcinomas and was potentially induced by IL-1α and IL-1β.^245,246^ In squamous cell carcinomas, MMP-13 is predominantly expressed on cancer cells and the stromal fibroblasts surrounding the cancer cells. In addition, MMP-13 is strongly indicative of the invasive and metastatic capacity of tumors.^247,248^ The specific role of MMP-13 has not yet been elucidated in breast cancer. A recent investigation revealed that the upregulation of MMP-13 in the tumor-stromal interaction, especially at the tumor-bone interface, is crucial to tumor-induced osteolysis, suggesting the potential value of MMP-13 in the treatment of breast cancers with bone metastasis.^249^

### Cancer cell adhesion and invasion

Among all the chemokines, SDF1a (also known as CXCL12) is particularly involved in bone metastasis^250^ and is often expressed in common metastatic sites such as BM. CXCR4 and CXCR7 represent two cognate receptors for CXCL12.^251–253^ Both CXCR7 and CXCL12 are highly expressed on certain cancer cells.^254,255^ CXCL12 can also be detected in normal tissues such as blood vascular endothelial cells,^255,256^ and fibroblasts are probably a major source of CXCL12 secretion in tumor tissue.^257,258^ Initially shown to facilitate the mobilization of hematopoietic stem cells and create a microenvironment for cancer stem cells,^259^ CXCL12/CXCR4 pathway signaling also plays an important role in cancer cell proliferation and angiogenesis.^259,260^

The inhibition of the CXCL12/CXCR4 interaction with CXCR4 mAb or CXCR4 blocking peptides prevents the migration of bone metastases of prostate cancer cells^261^ and reduces the in vivo metastatic load.^260^ Experimental evidence suggests that the CXCL12/CXCR4 signaling axis participates in prostate cancer cell adhesion to BM endothelial cells.^262–264^ Consistent with this hypothesis, antagonists of αvβ3, an adhesion-related integrin induced by CXCL12, significantly decreased the adhesion of prostate cancer cells to endothelial cells.^265^ CXCL12/CXCR4 is not the only signaling pathway involved in the adhesion of prostate cancer cells to endothelial cells but is instead part of a sequence of other interactions, such as those involving CD164.^266^

HER2/CXCR4/AKT signaling has been investigated in the bone metastasis of breast cancers.^267^ High expression of CXCR4 is found in breast cancer cells, promoting tumor cell homing and bone metastasis, with a highly osteolytic subclone observed in a breast cancer cell line.^268^ Addition of an anti-CXCR4 antibody or gene silencing of CXCR4 significantly decreased the migration of breast cancer cells to regional lymph nodes and the lung.^269^ Multiple preclinical studies have assessed the effectiveness of CXCR4 in blocking bone metastases in breast cancers.^269,270^ However, the CXCL12/CXCR4 axis can facilitate bone invasion processes by inducing MMP-9 and downregulating the expression of tissue inhibitor of metalloproteinases 2 in prostate cancer cells by this pathway.^271,272^ MMP family members are not only involved in cancer cell escape but also promote the extravasation of cancer cells from the ECM before proliferation in bones.^265^ Given that broad-spectrum MMP inhibitors fail to demonstrate clinical efficacy, more individualized targeting of proteinases may be a promising strategy to prevent bone metastases in cancer patients.

In addition to the CXCR4/CXCL12-induced CD164 and αvβ3 integrins mentioned above, a vast majority of adhesion molecules have been discovered in the interaction of cancer cells with BM endothelium. These molecules include galectin-3/Thomsen–Friedenreich antigen^273,274^ as well as CD44/hyaluronan,^275^ the inhibition of which impairs the adhesion of cancer cells to BM endothelial monolayers. Another member of the chemokine superfamily, CCL5, is produced by BMDCs and local stem cells in the bone microenvironment, and together with its receptor CCR5, CCL5 enhances cancer bone metastases.^276,277^ A recent report identified an increase in CCL5 secretion from bone stromal cells in the metastatic microenvironment, which induced prostate cancer cell migration involving androgen receptor signaling.^278^ The lymphotactin receptor (XCR1) is also a member of the chemokine receptor family, and its interaction with the ligand XCL1 substantially promotes the proliferation and migration of cancer cells.^279–281^ Other cancer cell-derived proteinases, such as ADAM^282^ and uPA82,^271^ are also implicated in the degradation of the bone matrix, promoting the effective invasion of cancer cells into bones. A recent study demonstrated the positive effect of cyclooxygenase-2 (COX-2) on cell adhesion and proliferation in bones.^283^ Human melanomas frequently overexpress the COX-2 gene,^284^ which exerts its regulatory effect on melanoma cell adhesion to proliferation in BMSCs in response to BMSC-derived VEGF.^283^

### Metastasis formation in bone

In addition to the processes by which cancer cells escape from original sites and invade bones, another fundamental step toward bone metastasis is the maintenance of cell proliferation as well as the consequent formation of metastases. As discussed above, the proposed mechanism for bone metastasis is the disruption of normal bone remodeling, leading to imbalanced bone resorption and bone formation. For cancer cell survival and growth, multiple growth factors produced by osteoblasts are embedded within the bone matrix and released during osteoclastic bone resorption.^285^

#### TGF-β

TGF-β participates in various normal physiological procedures, including immune responses and bone remodeling,^286^ and is also an important growth factor for osteoclastic bone resorption. Enhanced TGF-β signaling was detected in both preclinical^287–290^ and clinical breast cancer models.^288^ The TGF-β1 level was significantly elevated in the plasma of breast or prostate cancer patients with bone metastases.^291^ Smad-dependent TGF-β signaling was also observed in samples of the bone metastatic sites of breast cancer patients.^292^ However, TGF-β can play a paradoxical role in cancer, where it suppresses tumor growth at an early stage and promotes invasion and metastasis to bones at later stages.^293^ Various genes referred to as bone metastasis stimulators, including CXCR4, MMP-1, IL-11, JAG1, and PTHRP, were shown to be regulated by TGF-β,^288,294,295^ and anti-TGF-β therapies showed strong efficacy in controlling cancer-related bone metastases in mice.^289,296^

#### IGF

The IGF family is essential for bone growth,^297^ as all skeletal cells express IGF-1 and its receptor IGF-1R to maintain physiological functions.^297^ Moreover, IGF-1 promotes bone colonization of metastasizing tumor cells and facilitates their expansion inside bones. One such example is breast cancer metastasis stimulated by bone-derived IGFs through the activation of AKT and NF-κB to increase the proliferation and survival of cancer cells.^298^ In addition to the survival of cancer cells in bone, IGFs participate in the homing process. In triple-negative breast cancers, cancer-associated fibroblasts release IGF-1, which primes cells to home to the IGF-1-rich bone microenvironment.^299^ In prostate cancer with bone metastases, IGF-1 causes resistance-related genetic alterations of cancer cells through its binding to IGF-R, which has been identified in several proliferative and antiapoptotic mechanisms.^300^ This protective effect of IGF-1 on prostate cancer cells can be antagonized by agents that downregulate both local and systemic IGF-1 production, which is used to improve symptoms among prostate cancer patients with bone metastases.^301–303^

#### MicroRNA (miRNA)

To better adapt to the bone microenvironment, cancer cells undergo osteomimicry, which involves gene expression that is normally found on bone cells. The well-ordered expression of metastasis-related molecules during adaptation suggests that miRNAs are crucial regulators of osteomimicry. By binding to corresponding sequences in downstream target genes, miRNAs degrade and inhibit the translation of mRNAs.^304^ Accumulating evidence suggests that the aberrant expression of miRNAs indicates the invasive and metastatic phenotypes of tumor cells,^305–307^ and several miRNAs have been identified to mediate bone metastases, especially those of prostate cancer.^308–310^ MiR-141-3p is one of the earliest studied miRNAs. Previous studies found that the dysregulation of miR-141-3p is involved in the metastatic behavior of cancer cells.^311,312^ The expression of miR-141-3p disrupts NF-κB signaling by targeting TRAF5 and TRAF6, and the silencing of miR-141-3p promotes EMT, leading to cancer cell invasion and migration.^313^ Likewise, the downregulation of miR-145 caused by loss of wild-type p53 could promote bone metastasis through enhanced EMT.^314,315^ A growing number of miRNAs, such as miR-133b and miR-19a-3p, which directly target the activity of TGF-β signaling, have recently been shown to negatively regulate bone metastases of prostate cancers.^316,317^

Furthermore, the decreased expression of several miRNAs (e.g., miR-335, miR-126, and miR-206) was detected in human breast cancer cells metastasizing to the bone, and the restoration of their expression prevented bone metastatic progression.^318^ MiR-135 and miR-203 have been reported to downregulate the expression of Runx2 in breast cancer cells, thus reducing metastasis formation in bone.^319^ MiRNAs do not always exert negative effects on bone metastases, and some of them act as onco-miRNAs. The stimulation of miR-10b and miR-21, caused by the transcription factor Twist-1 and lysophosphatidic acid, respectively, promoted the invasion of breast cancer cells in BM.^320^

#### Autotaxin

Autotaxin (ATX or ENPP2) is a member of the nucleotide pyrophosphate–pyrophosphatase family. The expression of ATX in human primary breast tumor biopsies does not impact overall survival (OS), indicating that its expression at the primary tumor sites is not a prognostic indicator.^321^ However, a recent study showed that nontumoral ATX directs the early stage of tumor cell colonization in bones.^322^

## Management of cancer bone metastases

The optimal treatment of cancer bone metastases involves a multidisciplinary approach, including medical oncology, radiation oncology, and surgical oncology. Based on the basic biology of bone metastasis discussed above, concomitantly preventing new metastases and the growth of established metastases is theoretically an effective therapeutic strategy.^323^ The main treatment strategies for cancer bone metastasis are summarized in Table 2.

**Table 2:** The main treatment strategies for cancer bone metastasis

Therapy	Application	Examples
Hormone therapies		
SERM	Standard endocrine therapy for estrogen receptor-positive BC patients	Tamoxifen, raloxifene, and toremifene
AI	As monotheray or the extended adjuvant AI treatment after tamoxifen	Letrozole, anastrozole, and exemestane
Androgen deprivation therapy (ADT)	Standard treatment of PC patients with distant metastases	Antiandrogens, orchiectomy, GnRH agonists or antagonists
Radioisotopes		
β-emitting radioisotopes	Bone pain relief in PC patients	^89^Sr, ^135^Sm
α-emitting radioisotope	Treatment of CRPC patients with symptomatic bone metastases	^223^Ra
External beam radiation therapy (EBRT)	Prevents potential bone fractures and focal bone pain	
Surgery	Local adjuvant therapy combined with radiation therapy and embolization	Osteosynthesis and prosthetic implant insertion
Bisphosphonates		
Nitrogen-containing bisphosphonate (N-BP)	Prevents bone metastases in BC patients	ALN, ZOL, pamidronate
Non-nitrogen-containing bisphosphonates	Reduces bone metastases in BC patients during the 5-year follow-up	Clodronate, etidronate
Novel therapies		
RANK/RANKL inhibitor	Prevents bone metastases of BC and PC	Denosumab, OPG
CXCL12/CXCR4 inhibitor	Works in synergy with chemotherapy, radiation, or anti-VEGFR therapies	Anti-CXCL12 antibody
TGF-β inhibitor	Reduces tumor growth and bone metastases especially in triple-negative BCs	LY2109761, BMP7
HMGR inhibitor	Reduce osteolytic bone metastases of lung cancers	Simvastatin

*SERM* selective estrogen receptor modulator, *GnRH* gonadotropin-releasing hormone, *CRPC* castration-resistant prostate cancer, *ALN* alendronate, *ZOL* zoledronic acid, *AI* aromatase inhibitor, ^*223*^*Ra* radium-223, ^*89*^*Sr* strontium-89, ^*135*^*Sm* samarium-135, *BC* breast cancer, *PC* prostate cancer, *HMGR* HMG-CoA reductase

### Hormone therapies

Endocrine therapies are considered a first-line treatment for hormone-responsive cancer patients. For many years, tamoxifen, a selective estrogen receptor modulator (SERM), has been regarded as the standard endocrine therapy for patients with estrogen receptor-positive breast cancer. Based on clinical research, postsurgical tamoxifen treatment decreases breast cancer mortality by 34%.^324^ Other SERMs, including raloxifene and toremifene, have also been found to efficaciously block cell growth in patients with estrogen-responsive breast cancers.^325–328^ Aromatase is a key enzyme implicated in estrogen biosynthesis and converts androgens to estrogens. Recently, multiple large randomized trials have compared third-generation aromatase inhibitors (AIs), such as anastrozole, exemestane, and letrozole, with tamoxifen and found that AI therapies are more efficacious and tolerable in patients with breast cancer.^329–332^ Thus, AIs have now replaced SERMs in female cancer patients, especially postmenopausal women.^333–336^ Clinical treatment regimens include the upfront AI monotherapy,^329,337^ 2–3 years of tamoxifen prior to AI treatment,^338–340^ and extended adjuvant AI treatment.^341,342^ However, the AI-induced decrease in estrogen levels contributed to increased risks of bone resorption^343^ and fractures.^329,332,344^

Androgen deprivation therapy (ADT), a standard treatment for prostate cancer patients with distant metastases,^345,346^ can be achieved by antiandrogens, orchiectomy, and agonists or antagonists of gonadotropin-releasing hormone.^347^ The majority of ADT-treated patients experience symptomatic relief, metastasis regression, and decreased serum levels of PSAs. Similar to estrogen deprivation, ADT is also related to skeletal complications such as decreased bone mineral density and fracture risk.^348–351^ Abiraterone acetate is known to target CYP17A1, an essential enzyme for androgen synthesis, and substantially improved OS in prostate cancer patients with bone metastases.^352,353^ Similar results were obtained from another androgen receptor antagonist, enzalutamide, which is used as a first-line intervention for bone metastatic prostate cancer patients after castration and docetaxel treatment.^354,355^ However, most patients with prostate cancer eventually develop therapeutic resistance to androgen blockade, and it has recently been suggested that patients should receive at least three different lines of treatment.^356^

### Radioisotopes

Therapeutic radioisotopes with high affinity for bones such as phosphorus-32 have long been used to treat metastatic breast and prostate cancers.^357^ These radioisotopes can emit α- or β-particles and deliver detrimental radiation to cancer cells. The vast majority of radioisotopes harbor different physical properties, allowing them to address different clinical implications. The most commonly used β-emitting radioisotopes to treat bone metastases are strontium-89 (^89^Sr) and samarium-135 (^135^Sm). In one study, prostate cancer patients with bone metastases randomly received either ^89^Sr, a high-energy β-emitting radioisotope, or external beam radiation. Both types of treatment were effective, whereas the ^89^Sr treatment showed more effective relief of bone pain.^358^ Compared with ^89^Sr, ^135^Sm has a shorter half-life, allowing it to be delivered at larger doses with the same treatment time. A meta-analysis suggested that β-emitting radioisotope therapy alleviates bone pain over 1–6 months but frequently caused severe side effects, including leukopenia and thrombocytopenia.^359^ Moreover, ^89^Sr and ^153^Sm are renally excreted, which reduces their efficacy in patients with genitourinary cancers. Radium-223 (^223^Ra) is an α-emitting radioisotope abundant in the bone matrix in the area of osteoblast-induced mineralization. In a phase III trial, ^223^Ra prolonged OS in patients with castration-resistant prostate cancer and symptomatic bone metastases and was thus approved by the Food and Drug Administration in 2013.^360^ Ongoing clinical trials strive to optimize the treatment of bone metastases by evaluating the combination of radioisotopes with chemotherapy.^361–364^

### External beam radiation therapy (EBRT)

EBRT is a conventional palliative treatment for cancer bone metastases to prevent potential bone fractures and can function synergistically with surgical treatments. This treatment also provides timely control for focal bone pain, with ~50% of ERBT-treated patients reporting pain relief in 2 weeks.^200^ EBRT is effective even in radioresistant tumors, such as those originating from metastatic sarcoma or RCC.^365^ In the past few decades, multiple studies have compared the efficacy of high-dose, short-fraction radiation with radiation at lower doses and more fractions. Based on these trials, meta-analyses suggest no significant difference in either complete or partial responses between patients treated with hypofractionation or multifractionation EBRT.^366–368^ For example, in a recent study, similar pain-control effects were achieved with a single 8 Gy fraction and 30 Gy administered in ten fractions.^369^

### Surgery

One common complication of bone metastases is the large area of osteolytic lesions leading to high fracture risks, especially in breast and renal cancer metastases.^370^ In addition to bone pain as a result of fracture at any location, fracture of long bones such as the femur is more likely to cause disability and may decrease the quality of life and negatively affect prognosis.^371–375^ Prophylactic surgery for potential fracture includes plate osteosynthesis and prosthetic implant insertion.^376^ For maximum efficacy, radiation therapy and embolization are usually combined with surgeries as a local adjuvant treatment.^377,378^ Due to the limited guidelines for cancer bone metastases,^379,380^ many surgeons currently make clinical decisions based on the standard practice for fractures or according to their experience. A recent study provides an algorithm for the treatment of patients with long bone metastatic diseases, suggesting that the characteristics of individual bone lesions should be considered when performing surgical fixations or prosthetic reconstructions.^381^ Percutaneous cementoplasty is frequently adopted to treat bone metastases or to prevent impending fractures in weight-bearing bones. However, the single use of percutaneous cementoplasty is not necessarily curative and is suggested to be preceded by ablative treatment.^382^ Eisenberg et al. described a cervical cancer patient who received magnetic resonance-guided focused ultrasound surgery (MRgFUS) for iliac bone metastasis.^383^ In this case, MRgFUS led to a dramatic reduction in pain, and percutaneous cementoplasty was thus not considered. MRgFUS is a noninvasive surgical procedure that is effective in controlling bone metastasis-related pain in multiple clinical trials.^384–386^

### Bisphosphonates

Bisphosphonates are well known for their high affinity for the surface of bones that undergo bone resorption. Bisphosphonate therapy is commonly used for the long-term treatment of osteolytic and metastatic bone diseases. One classification of bisphosphonates is nitrogen-containing bisphosphonate (N-BP); for example, alendronate (ALN) and zoledronic acid (ZOL) are robust inhibitors of protein isoprenylation, which promotes osteoclast apoptosis. Examples of non-N-BPs include clodronate and etidronate, which induce osteoclast apoptosis by impairing mitochondrial function.^387,388^

In addition to the antiresorptive activity, the antitumor characteristics of bisphosphonates, including the inhibition of tumor cell adhesion and invasion,^390,391^ as well as induction of tumor apoptosis,^392^ have been extensively investigated both in vitro and in vivo.^389^ The antiangiogenic effect of bisphosphonates has been studied in several animal models.^393–396^ Pamidronate and clodronate can both abrogate angiogenesis in breast cancers, potentially by suppressing the expression of VEGF and the accumulation of IGF-1-induced HIF-1a protein.^397^ A recent study investigated connexin (Cx) 43 hemichannels, describing a self-defense mechanism of osteocytes against metastatic breast cancer cells.^398^ Treatment with either ALN or ZOL opens the Cx43 molecular passage between osteocytes and extracellular environments. In vivo studies also suggest that bisphosphonates could reduce tumor burden and bone metastasis formation in a dose-dependent manner.^399^

Randomized clinical trials evaluated the safety profile and the efficacy of pamidronate to prevent bone metastases in patients with breast cancer.^400,401^ The first skeletal-related event (SRE) was chosen as the primary outcome, and the number of SREs per year was recorded. The pamidronate-treated patients had fewer SREs per year and a longer time to the first SRE than those in the control group. In a phase II/III clinical study, patients with osteolytic lesions secondary to multiple myeloma or metastatic breast cancer were treated with pamidronate and ZOL to define the optimal dose of these two agents.^402,403^ The oral administration of clodronate prevented SREs.^404^ In female patients with primary breast cancer, the treatment of clodronate significantly reduced bone metastases during the 5-year follow-up.^405^ The oral administration of clodronate has also been reported to reduce both symptomatic progression and death in male patients with hormone-responsive diseases.^406^ ADT, as described above, may decrease the bone mineral density in prostate cancer patients and thus requires the application of bisphosphonates.^407^ Although osteoblastic bone metastases are frequently observed in patients with prostate cancer, the potent efficacy of bisphosphonates in these patients demonstrates the increased osteoclast activities in osteoblastic bone metastases.^408^ One of the side effects of bisphosphonates is osteonecrosis of the jaw (ONJ), which is associated with drug-exposed bone in the oral cavity.^409^ Due to the high dose of bisphosphonates in the treatment, more than 95% of patients with bone metastases present ONJ.^410^ In the case of ONJ, conservative treatments, including antibiotics and mouth rinses, are recommended.^411^

The combination of bisphosphonates and chemotherapy has also been studied. ZOL increased tumor cell apoptosis in vitro when administered after doxorubicin, possibly due to increased uptake of bisphosphonates caused by chemotherapy.^412^ A randomized phase II trial is currently testing the synergy of ZOL and 5-fluorouracil-epirubicin-cyclophosphamide in a neoadjuvant setting.^413^ Combined treatment with third-generation NBP (YM529) and IFN-α inhibited the aggravation of established bone metastases in the RCC model, which is probably mediated by the inhibition of YM529 on osteoclast activation and the antiangiogenic effect of IFN-α.^414^

### Novel therapies

**Fig. 3:**
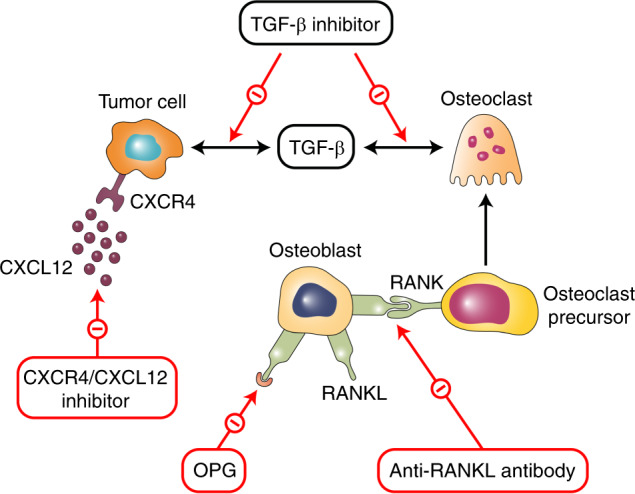
The target inhibition of pathways involved in bone metastases

Following the identification of the vicious cycle of bone metastases, novel agents that specifically target the complex pathways in bone metastases have been developed, many of which are currently under clinical evaluation. The target inhibition of pathways involved in bone metastases is presented in Fig. 3.

RANK/RANKL signaling is an essential strategy for blocking targets for osteolytic bone diseases. Denosumab, a humanized RANKL antibody and the first drug of this class, has demonstrated superiority over ZOL in preventing bone diseases in both breast and prostate cancers with bone metastases.^415,416^ Denosumab prevents the RANKL/RANK interaction by mimicking the action of OPG and thus reduces the survival and bone resorption activity of these osteoclasts.^417,418^ Based on the overall satisfactory results of clinical trials, denosumab has now been approved for SRE prevention in patients with cancer. A recent study found that AMG161, an equivalent of denosumab, could block RANKL signaling and the formation of micrometastases in BM.^419^

As previously discussed, the CXCL12 pathway is an important regulator of metastases in prostate, colorectal, and breast cancers. Blockade of the CXCL12 pathway has been found to substantially delay both primary tumor growth and distant metastasis in multiple preclinical studies.^137–141^ Anti-CXCL12 agents work especially well in the prophylactic setting when treatments start early and are less effective in established tumors.^420^ A previous report showed that the pan-VEGFR tyrosine kinase inhibitor cediranib could upregulate circulating CXCL12 concentrations,^421–424^ and genetic testing results revealed that CXCL12/CXCR4 pathway activation could lead to the specific inhibition of VEGFR activity in BMDCs.^425^ Therefore, anti-CXCL12 therapy can be used in combination with anti-VEGFR therapies to reach maximum clinical efficacy. Other anticancer treatments are also promising candidates for anti-CXCL12 agents. Several chemotherapeutics, such as paclitaxel or vascular-disrupting agents, can lead to an increased level of circulating CXCL12 and mobilization of BMDCs.^426,427^ Moreover, irradiation upregulates CXCL12 expression, both directly and indirectly, through hypoxia and the related HIF-1α activation.^428–430^ These results indicate that when used in synergy with other treatment options, anti-CXCL12 therapy demonstrates promising efficacy compared with monotherapy.

TGF-β inhibitors represent another class of novel therapies to prevent cancer bone metastases, as more than half of breast cancers display increased TGF-β activities.^431^ The inhibition of TGF-β has been found in preclinical research to suppress tumor growth and distant metastases, including those to bones, and is highly potent in triple-negative breast cancers.^432^ Many TGF-β antagonists, most of which remain at the preclinical stage, are currently under clinical development. These TGF-β antagonists include TGF-β antibodies (1D11),^433–435^ receptor kinase inhibitors such as LY2109761,^249,389,390^ and other antagonizing agents such as BMP7.^389,390^

HMG-CoA reductase (HMGR) inhibitors have been examined in different cancers (e.g., lung cancer).^436^ HMGR is an important enzyme for cholesterol biosynthesis,^437^ the inhibition of which has demonstrated antitumor effects in multiple tumor types. Simvastatin is an HMGR antagonist and can reduce osteolytic bone metastases of lung cancers, potentially through the downregulation of CD44, P53, and MMPs^438^ or the antagonistic interaction between p53 and CD44.^439^ Furthermore, a thrombin inhibitor, argatroban, could reduce the bone metastasis of breast cancer cells by suppressing the activation of tissue factors and VEGF secretion.^440^ The ET axis is another promising therapeutic target for the treatment of prostate cancer bone metastases. In clinical studies, the ETAR inhibitor atrasentan successfully decreased PSA in male patients with hormone-refractory disease^185^ and markers of bone turnover in prostate cancer patients with bone metastases.^441^

Biological intracontrol treatment (BICT) is an herbal medicine-based therapy involving herbal extracts and palliative care. In a case report, a 59-year-old lung cancer patient who failed first‑line chemotherapy treatment and presented with multiple bone metastases was concomitantly treated with BICT and bisphosphonates, which inhibited tumor growth and simultaneously promoted bone repair.^442^ Fish oil has been shown to have a new function, targeting the prometastatic molecule CD44 on the cell surface to suppress the migration and invasion of tumor cells.^443^ Tasquinimod is an experimental drug that has been proven effective in controlling both tumor growth and distant metastases of prostate cancer.^444–448^ The methyl group donor is another drug under experimental evaluation. S-adenosylmethionine has also been found to reduce skeletal metastases both in vitro and in vivo, which probably correlates with increased bone density.^449^ Since bone loss is a severe complication of cancer patients with bone metastases, various bone-anabolic agents that stimulate the synthetic activities of osteoblasts, such as PTH agents, are commonly used in clinical practice. Examples of anabolic agents to prevent bone loss include CaSR antagonists^450–452^ and PTH/PTHrP.^453,454^

Hypoxic signaling contributes to the preparation of the bone microenvironment for cancer metastases and is therefore an attractive therapeutic target. The inhibition of the hypoxia pathway impairs the development of chemotherapeutic resistance mediated by HIF. A wide variety of hypoxic signaling inhibitors, including the small molecule inhibitor (SMI) 2-methoxyestradiol,^455^ which downregulates HIF-1α levels and VEGF expression in tumor cells, are under preclinical investigation.^456,457^ Clinical evaluations have also been initiated to assess 2-methoxyestradiol and analogs to treat multiple cancers, with the analogs exhibiting more potent antiangiogenic and antitumor effects.^458^ Other examples of SMIs that target hypoxic signaling include inhibitors of topoisomerases I and II as well as PI3K inhibitors, which negatively act on HIF-mediated gene transcription.^459^ Based on the interaction between HIF-1 and many other signaling pathways, inhibitors of hypoxic signaling may be used in combination with other therapies to induce sufficient suppression of tumor growth and spread.^460^

## Conclusion and perspective

Bone metastasis is one of the most lethal complications of cancer, and further elucidation of this process should provide new insights into the bone tropism of cancer cells and novel therapies that reduce mortality in cancer patients. Three steps contributing to bone metastases include (1) cancer cell escape and dissemination, (2) adhesion and invasion to bone, and (3) colonization and metastasis in bone. The first step is similar to metastases to nonbone organs, such as the lung and liver, whereas the second and third steps are specific to bone metastases, owing to their distinct cellular and molecular profiles. In particular, bone metastasis involves complex interactions between tumor cells and the bone microenvironment. Thus, the various pathways in the bone microenvironment, such as RANK/RANKL signaling, can be specific therapeutic targets for bone metastases.

In addition to traditional treatments such as hormone therapies, radioisotopes, and bisphosphonates, novel inhibitors that block these pathways, such as the RANKL inhibitors that block osteoclast differentiation, have demonstrated significant antitumor effects in bone metastasis models. It is expected that the concomitant use of novel inhibitors with conventional therapies will provide optimal treatment for bone metastases, but long-term clinical studies are needed to evaluate whether these combinations lead to survival benefits in patients. An in-depth elucidation of the premetastatic niche in bone is also essential for the early intervention of bone metastases. However, the majority of studies on the premetastatic bone niche are based on animal models, which may not fully represent the bone microenvironment in humans. Developing animal models that can mimic the general bone metastatic process in human cancers is thus essential. Moreover, elucidating the specific mechanisms for bone metastases in diverse tumors will promote the development of tumor-type-specific treatments for bone metastases. Hopefully, the current knowledge and ongoing studies will provide additional alternatives for the treatment of cancer patients with bone metastases.
